# Supplementary material for: Hyperspectral imaging as an objective diagnostic tool for secondary lymphedema in breast cancer patients
Source: Commun Med (Lond). 2025 Dec 18;6:45. doi: 10.1038/s43856-025-01301-y (PMC12823606; doi:10.1038/s43856-025-01301-y)
Supplement: Supplementary file 2 — Supplemental Information [file 43856_2025_1301_MOESM2_ESM.pdf]

Supplementary figures

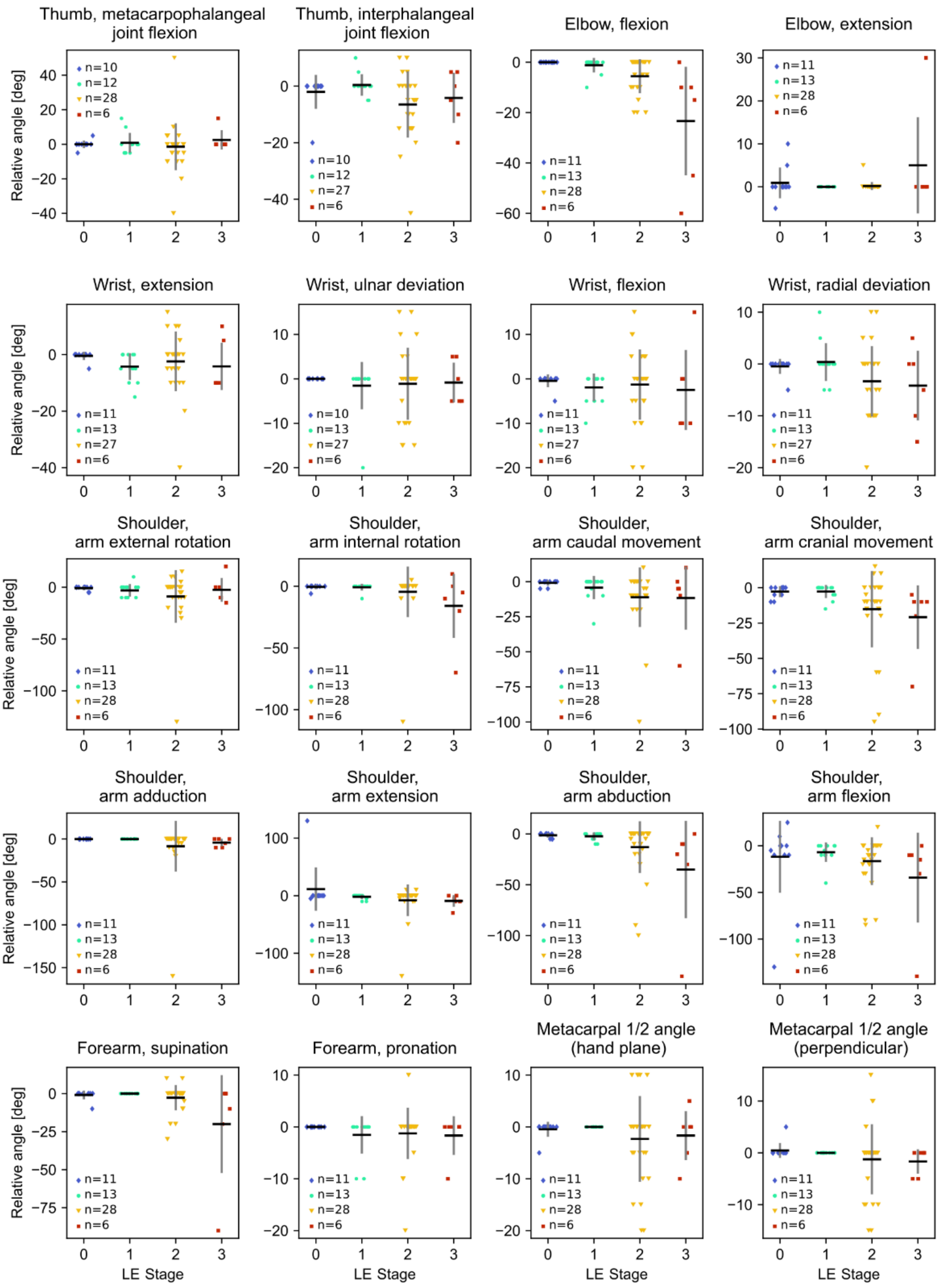

Figure S1. Difference in movement angle between the affected and unaffected sides, measured at the fingers, arm and shoulder. Each point represents data from one patient. Horizontal lines indicate the means, while vertical bars represent the standard deviation of the distributions. Sample sizes are show in each panel.

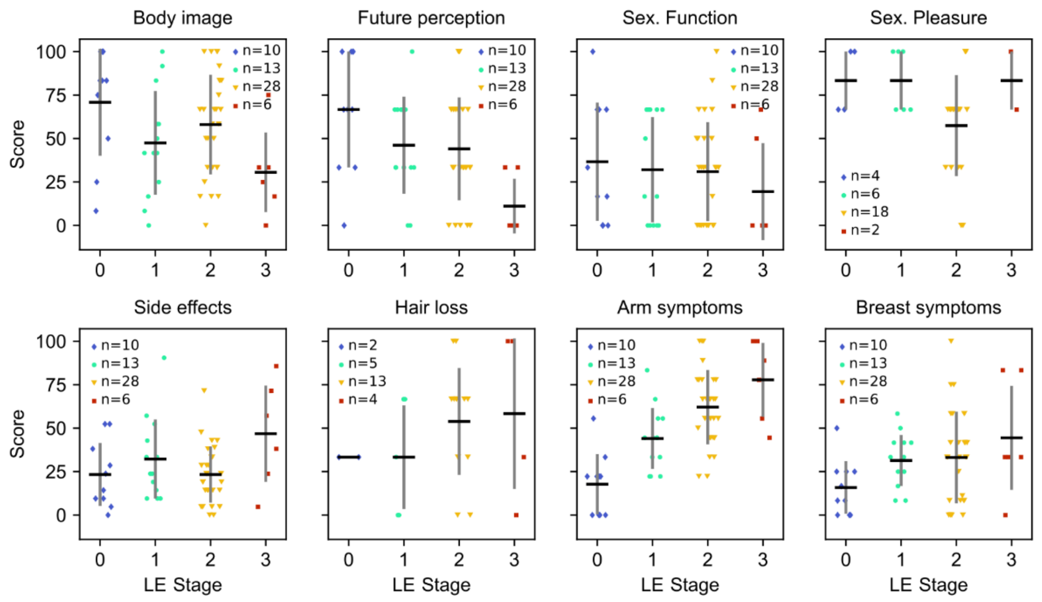

Figure S2. Patient-reported outcomes as assessed by the Lymph-ICF-UL and EORTC QLQ-BR23 questionnaires. For categories related to self-perception and function (e.g., body image, future perspective, sexual functioning), higher scores indicate a positive self-image and greater level of functioning. Conversely, for symptom-related categories (e.g., side effects, arm and breast symptoms, hair loss), higher scores reflect greater symptom severity. Horizontal lines indicate the means, while vertical bars represent the standard deviation of the distributions. Sample sizes are show in each panel.
