# Supplementary material for: Hyperspectral imaging as an objective diagnostic tool for secondary lymphedema in breast cancer patients
Source: Commun Med (Lond). 2025 Dec 18;6:45. doi: 10.1038/s43856-025-01301-y (PMC12823606; doi:10.1038/s43856-025-01301-y)
Supplement: Supplementary file 3 — Description of Additional Supplementary Files [file 43856_2025_1301_MOESM3_ESM.docx]

**Description of Additional Supplementary Files**

Supplementary Data 1: Source data for Figure 3 and 4 i,j

Supplementary Data 2: Source data for Supplementary Figures S1 and S2
